# The Impact of Antioxidant Supplementation and Heat Stress on Carcass Characteristics, Muscle Nutritional Profile and Functionality of Lamb Meat

**DOI:** 10.3390/ani10081286

**Published:** 2020-07-28

**Authors:** Surinder S. Chauhan, Frank R. Dunshea, Tim E. Plozza, David L. Hopkins, Eric N. Ponnampalam

**Affiliations:** 1 School of Agriculture and Food, Faculty of Veterinary and Agriculture Sciences, The University of Melbourne, Parkville, VIC 3010, Australia; ss.chauhan@unimelb.edu.au (S.S.C.); fdunshea@unimelb.edu.au (F.R.D.); 2 Faculty of Biological Sciences, The University of Leeds, Leeds LS2 9JT, UK; 3 Chemistry, Agriculture Victoria Research, Department of Jobs, Precincts and Regions, Macleod, VIC 3085, Australia; Tim.Plozza@agriculture.vic.gov.au; 4 NSW Department of Primary Industries, Centre for Red Meat and Sheep Development, P.O. Box 129, Cowra, NSW 2794, Australia; david.hopkins@dpi.nsw.gov.au; 5 Animal Production Sciences, Agriculture Victoria Research, Department of Jobs, Precincts and Regions, Bundoora, VIC 3083, Australia

**Keywords:** aged meat, animal performance, fatty acids, linoleic acid, oxidative stress, sheep

## Abstract

**Simple Summary:**

The increased incidence of heat stress in lambs has been reported in many countries, especially warmer parts of the world, compromising lamb welfare, having flow on effects for meat quality traits. While heat stress impacts can be variable depending on the severity and duration, the exposure of lambs to one week of elevated temperature increased the muscle omega-6 fatty acid concentration compared with the thermoneutral group. The one week heat stress is believed to enhance pro-inflammatory actions through induced free radical formation and oxidative stress. Somewhat independently, supplementation of the diet with vitamin E improved the growth rate and reduced oxidative stress. This suggests that under more extreme heat stress conditions, lambs fed in feedlots would benefit from enhanced levels of antioxidants such as vitamin E.

**Abstract:**

The impact of antioxidant supplementation and short-term heat stress on lamb body weight gain, meat nutritional profile and functionality (storage stability of lipids and colour) of lamb meat was investigated. A total of 48 crossbred ((Merino × Border Leicester) × Dorset) lambs (42 ± 2 kg body weight, 7 mo age) were randomly allocated to three dietary treatments (*n* = 16) by liveweight (LW) that differed in dosage of vitamin E and selenium (Se) in the diet. Vitamin E and Se levels in the control (CON), moderate (MOD) and supranutritional (SUP) dietary treatments were 28, 130 and 228 mg/kg DM as α-tocopherol acetate and 0.16, 0.66 and 1.16 mg Se as SelPlex™/kg DM, respectively. After four weeks of feeding in individual pens, including one week of adaptation, lambs were exposed to two heat treatments. Animals were moved to metabolism cages for one week and subjected to heat treatments: thermoneutral (TN; 18–21 °C and 40–50% relative humidity) and heat stress (HS; 28–40 °C and 30–40% relative humidity) conditions, respectively. Final LW and hot carcass weight were influenced by dietary treatments with higher final live weight (FLW) (*p* = 0.05; 46.8 vs. 44.4 and 43.8 kg, respectively) and hot carcass weight (HCW) (*p* = 0.01; 22.5 vs. 21.3 and 21.0 kg, respectively) recorded in lambs fed the SUP as opposed to the CON and MOD diets. Vitamin E concentration in the longissimus lumborum (LL) muscle tended to be higher in lambs fed MOD or SUP diets than the CON group. Lipid oxidation of aged meat at 72 h of simulated retail display was reduced by antioxidant supplementation. Short-term (one week) heat stress treatment significantly increased muscle linoleic acid and total omega-6 concentrations compared with the CON group. The results demonstrate that four-week antioxidant supplementation at the SUP level improved animal productivity by increasing LW and carcass weight and the functionality of meat exhibited by reduced lipid oxidation. An increase in muscle omega-6 fatty acid concentration from short-term heat stress may induce oxidative stress via proinflammatory action.

## 1. Introduction

Studies into global warming and increased climate variability show the potential threat for food security in the coming decades [1,2]. Therefore, plant (crop and pasture) and animal production systems must adapt to manage variable weather patterns and increases in the frequency of extreme weather conditions. Long-term elevated temperatures and low rainfall during summer-autumn seasons may affect pasture and crop persistence, yield and nutrient composition. When seasonally available feeds are in short supply such as pasture and fodders, animals must be fed with specialised feeds, forages and supplements to ensure that nutrient requirements, health and productivity are not compromised [3].

Elevated temperature and extreme hot conditions have been reported to cause heat stress, which in turn compromises the metabolic status and antioxidant defence systems of animals, leading to reduced performance and productivity [4]. Under heat exposure, an animal’s body temperatures can rise beyond the thermoneutral zone to the heat load zone, and when total heat load exceeds the animal’s capacity to dissipate heat, animals are subject to heat stress. Such conditions can interfere with animal antioxidant defence systems, with tolerance to stresses ultimately causing illness and/or productivity loss [5,6]. Productivity loss in animals is mainly due to reduced feed intake, the consumption of feeds low in nutrients and the loss of energy due to regulation of body temperature (thermoregulation). Based on the geographic location and temperature regions where the studies are undertaken in farm animals, the thermoneutral zone ranges have been categorised differently by various researchers; for example, in dairy cattle, 5 to 15 °C as proposed by Hahn et al. [7] and 5 to 25 °C by Roenfeldt [8]. Therefore, the magnitude and type of changes in animal metabolism (as indicated by DNA damage, protein denaturation or lipid peroxidation), as well as loss in quality (e.g., meat colour) and quantity (e.g., liveweight or carcass weight) are expected to deviate for different temperature zones and different demographic regions. 

In recent years in Australia, there has been an increase in the incidence of heatwaves, leading to increased exposure of animals to high environmental temperatures along with prolonged drought, exposing animals to heat stress conditions. Heat stress can induce oxidative stress and metabolic disorders by elevated levels of free radicals and/or reactive oxygen species in the circulatory system if the antioxidant defence system is depleted [9]. This can result in the peroxidation of lipids and may compromise the nutrient composition (essential fatty acids) and storage stability (retail colour and lipid oxidation) of meat. Poor feed intake and health status associated with heat stress can cause economic losses not only due to a decline in animal productivity, but also due to a reduced muscle quality (colour, lipid oxidation) [9,10]. With the growing concern for animal welfare and consumer awareness of food quality, there has been increased focus on improving the nutritional value and shelf life of aged meat. Vitamin E is known to improve the antioxidant capacity of muscle and hence meat quality by avoiding/delaying the lipid peroxidation, protein oxidation and discoloration of muscle meat [11]. This study investigated the effect of short-term heat stress and vitamin E supplementation on carcass traits, meat nutritive value and the storage stability of meat (lipids and colour) in sheep. We hypothesised that heat induced oxidative stress can cause changes in polyunsaturated fatty acids, while antioxidant supplementation can improve animal performance and/or the lipid storage stability of meat.

## 2. Materials and Methods

Animals were cared for and handled as per standard procedures for the handling and care of live animals for research purposes following the guidelines of the Australian Code of Practice for Care and Use of Animals for Scientific Purposes. All procedures undertaken in this study were approved by The University of Melbourne Science, Optometry and Vision sciences and the Land and Environment Animal Ethics committee, AEC No # 1312892.1.

An animal feeding study was conducted at the sheep research facilities, University of Melbourne, Dookie campus. The experimental design, dietary treatments and measures of blood intermediary metabolites were reported earlier [4]. Briefly, forty-eight crossbred ((Merino × Border Leicester) × Dorset) lambs (42 ± 2 kg body weight, 7 months age) were randomly allocated to one of three diets; based on antioxidant doses of vitamin E and Se in the diet, control (CON), moderate (MOD) and supranutritional (SUP); and two temperature treatments (thermoneutral and heat stress) in a 3 × 2 factorial design. A standard finisher pellet ration (17% CP and 3.0 Mcal ME/kg DM) was used as the basis of treatment diets. The vitamin E and Se concentrations in the CON, MOD and SUP dietary treatments were 28, 130 and 228 mg/kg DM as α-tocopherol acetate and 0.16, 0.66 and 1.16 mg Se as SelPlex™ kg/DM, respectively. Lambs on each dietary treatment were fed in individual pens for 4 weeks with 1 week of adaptation and then moved to metabolism cages for 1 week of short-term cyclic heat stress. The lambs on each dietary treatment were randomly allocated to either thermoneutral (TN; 18–21 °C and 40–50% relative humidity) or heat stress (HS; 28–40 °C and 30–40% relative humidity) conditions in the purpose-built climatic chambers where heat was turned on at 9 a.m. (allowing the temperature to rise to the peak at 40 °C in 4–6 h and then maintained between 38 and 40 °C till 5 p.m.) and turned off at 5 p.m. (allowing the temperature to drop to 28 °C by 6 p.m. and then maintained between 26 and 28 °C overnight till the next morning at 9 a.m.).

At the end of the feeding and heat treatment, the lambs were transported (200 km) to a commercial abattoir (Brooklyn, VIC, Australia) and kept in lairage overnight for 14 h. The following morning at 6 a.m., physiological parameters (respiration rate and rectal temperature) were recorded from all lambs and a blood sample collected as reported by Chauhan et al. [4]. The lambs were slaughtered by stunning, and carcasses were electrically stimulated at 30 min post-slaughter. Hot carcass weight (HCW) and GR fat depth were recorded at 1 h post-slaughter where GR is the total tissue thickness over the 12th rib, 110 mm from the backbone, which is measured with a GR knife. The carcasses were chilled overnight. At 24 h post-slaughter, the muscle longissimus lumborum (LL) was dissected from the left side of the carcass between the 9th and 10th lumbar vertebrae to the caudal end, trimmed of external fat and connective tissue and muscle samples collected for meat quality evaluation. The ultimate pH (pH at 24 h) was recorded in the muscle LL using a portable pH meter with temperature compensation (WP-80, TPS Pty Ltd., Brendale, QLD, Australia) and a polypropylene spear-type gel electrode (Ionode IJ 44, Tennyson, QLD, Australia), calibrated at chiller temperature. For antioxidant (vitamin E) and fatty acid composition determination, samples were stored at −20 °C until further analysis.

Muscle samples were vacuumed packed and stored for 6 weeks at 2 °C to evaluate aged meat retail colour stability. For retail colour stability, two slices of a 2 cm thickness of LL muscle were placed on a black foam tray and over wrapped with a 15 μm thickness PVC food film. Both fresh and aged meat from the LL muscle were sliced at Day 1 and Day 42 post-slaughter, respectively, and maintained at 3–4 °C under fluorescent light (1000 lux) for 72 h. Redness (a*-value) and brownness formation (RF_630/580_) of meat were recorded two times on each sample using a Hunter Laboratory Mini Scan XE Plus meter (Model 45/0-S, Hunter Associates Laboratory Inc., Reston, VA, USA) with a 25 mm aperture (light source set to illuminant D-65 and a 10 degree standard observer) as described by Ponnampalam et al. [12]. The measurements were taken at 1 h (Day 0), 24 h (Day 1), 48 h (Day 2) and 72 h (Day 3) of retail display. The percentage of light reflectance at a wavelength of 630 nm divided by the percentage of light reflectance at a wavelength of 580 nm (RF_630/580_) is an estimate of the oxymyoglobin/metmyoglobin myoglobin ratio, which was used as an indication of brownness formation on the meat surface [13]. 

Muscle fatty acid composition was determined using the method described by Ponnampalam et al. [12]. Briefly, zero-point-five grams of homogenised, freeze-dried muscle tissue were spiked with 100 μL of 10 μg/mL C19:0 internal standard, then hydrolysed in a mixture of 0.7 mL of 10 N KOH in water and 5.3 mL of methanol with incubation at 55 °C for 1.5 h. Fatty acid methyl esters (FAME) were then produced by the addition of 0.6 mL of 24 N sulfuric acid in water and further incubation as above. The FAME were partitioned into 1 mL of hexane and 1μL injected into a Varian 3800 GC with FID detector and 100 m × 0.25 mm Varian CP-Sil 88 column (Varian, Mulgrave, VIC, Australia). FAME peaks were identified and quantified using a reference standard (Supelco C4-C24 mix, Sigma Aldrich, St. Louis, MO, USA) to which the C19:0 internal standard had also been added. Fatty acid concentrations in meat were presented as mg/100 g of fresh sample according to Food Standards Australia & New Zealand (FSANZ) nutrition guidelines of foods.

Vitamin E concentration of both experimental diets and muscle tissue was determined by reversed phase HPLC using a method derived from Ball [14]. Briefly, two grams of feed or fresh muscle were saponified in a mixture of 40 mL ethanol, 0.5 g ascorbic acid and 10 mL 1:1 KOH in water, at reflux. This solution was then extracted 3 times with hexane containing butylated hydroxytoluene and a suitably sized aliquot of the combined extracts evaporated to dryness under a flow of nitrogen. The residue was redissolved in 1 mL of methanol, filtered through a 13 mm 0.45 μm Teflon filter disc and 20 μL injected into a Waters 2695 HPLC (Waters, Milford, MA, USA) fitted with a 3.9 × 300 mm, 10 μm Bondclone C18 column (Phenomenex, Sydney, Australia). The mobile phase consisted of water:methanol (5:95), at a flow rate of 1 mL/min. Vitamin E was selectively detected using a Shimadzu RF-10Axl fluorescence detector (Ex 295 nm, Em 330 nm) and quantified by the comparison of the peak area with a series of standards prepared from neat vitamin E (Sigma-Aldrich, St. Louis, MO, USA).

Lipid oxidation in fresh and aged meat collected at 1 h (Day 0) and 72 h (Day 3) of retail display was determined by measuring the concentration of malondialdehyde (MDA, expressed in mg/kg of muscle) using the thiobarbituric acid reactive substances (TBARS) procedure [15]. Approximately 10 g samples were homogenised for 45 s with 30.0 mL of chilled extraction buffer that contained 20% trichloroacetic acid (TCA) and 2 M phosphoric acid. An additional 30.0 mL of chilled water was added before the solution was homogenised for a further 15 s and then filtered through Whatman No. 1 filter paper. Filtrate aliquots (2.0 mL) were mixed with 2.0 mL of 2-thiobarbituric acid (5 mM) and held overnight (~12 h) under darkness at room temperature. The wavelengths of standards and samples were measured using a Carey 300 spectrophotometer with a sipper attachment (set sipper intake 3 mL in 10 s) and wavelength set at 532 nm.

### Statistical Analysis

The data were analysed using the GenStat statistical package (14th Edition, VSN International Ltd., Hemel Hempstead, UK). The experimental design was 2 × 3 factorial with two levels of temperature treatments (heat stress and thermoneutral) and 3 levels of dietary antioxidant treatments (CON, MOD and SUP). Final live weight (FLW), HCW and GR fat depth was adjusted to the initial LW of the lambs. The variables of carcass traits, muscle fatty acids and vitamin E concentrations and lipid oxidation were tested for the significance of the main effects and the interactions of temperature treatments by dietary antioxidant treatments. The effect of dietary and temperature treatments on meat colour traits (redness; Hunter Lab a*-values and brownness formation; Hunter Lab reflectance at 630/580) and lipid oxidation (TBARS; MDA equivalents) over the display time were analysed using display time points as repeated measurements using an ANOVA. *F*-tests were used to determine the overall significant difference among the predicted means at *p* ≤ 0.05, but all the values are reported in the tables with results.

## 3. Results

There was an effect (*p* = 0.05) of diet on the final live weight (FLW) and HCW, and the lambs on the SUP diet showed greater FLW (46.8 vs. 44.4 and 43.8 kg, respectively) and HCW (22.5 vs. 21.3 and 21.0 kg, respectively) than lambs fed the CON and MOD diets (Table 1). There was an effect (*p* = 0.01) of diet on carcass fatness as assessed by GR such that lambs fed the MOD diet had lower carcass fatness compared with control and SUP groups (8 vs. 9.8 and 10 mm for moderate, control and SUP, respectively). However, there was no effect of heat treatment (heat stress) or the interaction of diet by heat treatment on FLW, HCW or GR fat depth. Muscle LL vitamin E content tended to be higher (*p* = 0.15) with the antioxidant supplementation such that lambs supplemented with MOD or SUP antioxidants had a greater concentration compared with CON lambs (Table 1). 

There was no effect of heat stress on muscle vitamin E concentration or any interaction between heat stress and antioxidant supplementation, nor on muscle fatty acid concentrations, except for linoleic acid (LA) and total n-6 (Table 2). One week of thermal stress increased (*p* < 0.03) on average muscle LA (85 vs. 92 mg/kg), which subsequently led to increased (*p* = 0.02) total n-6 concentration (107 vs. 114 mg/kg;) compared with the TN group (Table 2).

**Figure 1:**
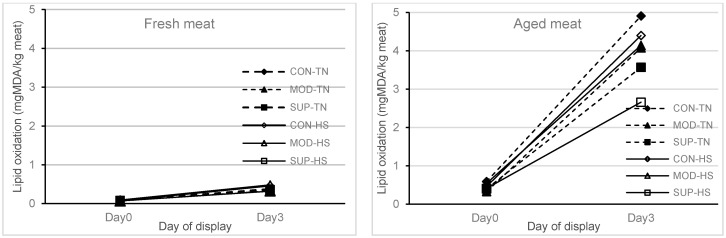
Lipid oxidation (malondialdehyde (MDA), mg/kg meat) in fresh and aged (packaged and stored for 42 days at 2 °C refrigerated condition) lamb meat (muscle longissimus lumborum (LL)) at 1 h (Day 0) and 72 h (Day 3) of simulated retail display. Lambs were finished on four weeks of experimental diets ^1^ followed by one week thermal treatment ^2^. ^1^ Control (CON) = lambs supplemented Vit E @ 28 mg/kg DM and Se @ 0.16 mg/kg DM; moderate (MOD) = lambs supplemented Vit E 130 mg/kg DM and Se @ 0.66 mg/kg DM; supranutritional (SUP) = lambs supplemented Vit E @ 227.5 mg/kg DM and Se @ 1.16 mg/kg DM. ^2^ Thermal treatment = lambs housed in either a thermoneutral (TN) room with temperature ranging from 18–21 °C and relative humidity (RH) ranging from 40–50% or a heat stress (HS) room with temperature ranging from 28–40 °C and RH ranging from 30–40%, for one week followed by transportation to the abattoir and a 14 h lairage period.

**Figure 2:**
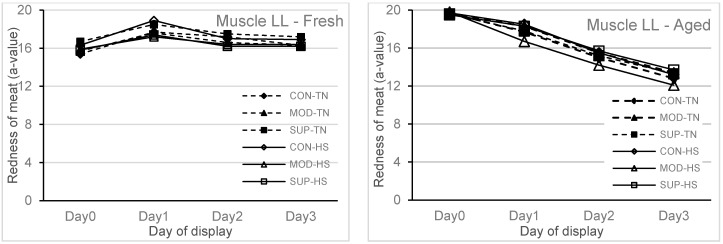
Redness (a-value) of fresh and aged (packaged and stored for 42 days at 2 °C) meat (muscle LL) assessed at 1 h (Day 0), 24 h (Day 1), 48 h (Day 2) and 72 h (Day 3) of simulated retail display condition (3–4 °C) from lambs fed four weeks of experimental diets and followed by one week of thermal treatment. See Figure 1 for an explanation of the labels.

**Figure 3:**
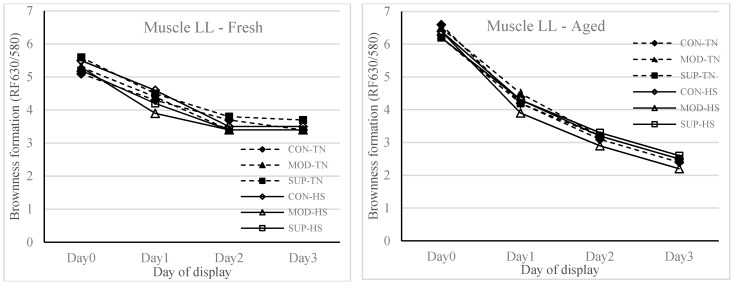
Brownness formation (reflectance 630/580) of fresh and aged (packed and stored for 42 days at 2 °C) meat (muscle LL) assessed at 1 h (Day 0), 24 h (Day 1), 48 h (Day 2) and 72 h (Day 3) of simulated retail display condition (3–4 °C) from lambs fed four weeks of experimental diets and followed by one week of thermal treatment. See Figure 1 for an explanation of the labels.

In the current study, one week of exposure to HS did not affect the lipid oxidation of fresh and aged (stored for six weeks) meat that was displayed under refrigerated conditions for 72 h compared to the TN group. At the same time, there was an effect (*p* = 0.05) of diet on the lipid oxidation of aged meat, and the reduced lipid oxidation of aged meat at 72 h display was reduced in the SUP group as compared to the CON group (Figure 1). There were no interactions of diet × heat treatment × meat type × display time or diet × heat treatment × meat type or diet × meat type × display time or heat treatment × meat type × display time for any of the colour traits analysed. Therefore, results are reported separately for fresh and aged meat (type of meat) over the 72 h display time, as shown in Figure 2 (redness of meat) and Figure 3 (brownness formation), respectively. Meat type (fresh vs. aged) × display time (1, 24, 48 and 72 h) also showed an effect (*p* < 0.001) on redness and brownness formation. The redness was higher and brownness formation was lower at 1 h display for aged meat and showed a rapid decline during 48 to 72 h display as compared to fresh meat. The values for redness and brownness formation in fresh meat at 48 h of display were greater (*p* < 0.05) than the values obtained for aged meat at 48 h of display. 

## 4. Discussion

Environmental challenges such as extreme weather events (e.g., high temperature) and nutritional deficiencies (e.g., low availability of green pasture) can affect animal health and wellbeing, as well as productivity. Heat stress has been implicated in oxidative stress in farm animals during hot summer conditions [6,16]. The dynamics of the heat stress can vary with the geographical zone, but the resilience and adaptability to thermal stress of an animal may vary with individuals based on the physiological status, metabolic demand for production and the antioxidant potential of the body [10,12]. 

Human and animal studies report that increased dietary intake of antioxidants can protect the cellular components of DNA, lipids and proteins from reactive oxygen species [17,18], yielding lower levels of free radical substances and other secondary metabolites in the body. Dietary antioxidant supplementation thus minimises oxidative stress in animals [19,20], leading to better growth performance associated with improved feed efficiency or nutrient utilisation in the body, as evidenced by increased liveweight and carcass weight in SUP compared with MOD and CON treatments. We also observed that vitamin E and Se supplementation at the SUP level tended to increase muscle vitamin E concentration compared with the CON group. These findings are similar to a previous study [21], which showed an increase in carcass weight and muscle vitamin E content of lambs grazed on senesced perennial (lucerne) pastures as compared to lambs finished on annual pastures with some cereal or oilseed supplements. The latter indicates that the application of lucerne in ruminant (sheep and cattle) diets may potentially improve tissue vitamin E concentration (antioxidant status) when roughage or feedlot diets without supranutritional level of vitamin E and Se are provided.

Food from some edible wild plants provides a good balance of omega-3 and omega-6 fatty acids. Secondary mediators of dietary omega-3 and omega-6 fatty acids have anti- and pro-inflammatory effects, respectively, in animals and humans. The application of grain based high energy diets in modern agriculture to induce faster growth and greater yield in livestock has decreased the omega-3 content of animal foods such as meat, eggs and milk, leading to an unhealthy omega-6 to omega-3 ratio of 16–20:1 instead of 1–4:1 reported in the past [22]. This has been proposed as the reason for several inflammatory diseases, propagated by oxidative stress and metabolic disorders [22]. Muscle LA concentration was increased following heat treatment of lambs, which resulted in higher muscle n-6 content than the lambs reared in TN conditions. The LA and total n-6 concentrations in the LL muscle also tended to be higher in lambs fed the CON diet, and lambs fed antioxidants at supranutritional levels had a lower concentration compared to control lambs. This implies that even a short period of heat stress, i.e., one week of heat exposure to lambs, can significantly alter the muscle n-6 fatty acid content, possibly through the changes in lipid biosynthesis in the muscle tissue systems. This may have implications for animal health (glucose intolerance, pro-inflammatory actions) and muscle quality (lipid oxidation and colour stability of meat) due to the alteration of the muscle membrane structure, fluidity or permeability.

Previously, feeding a concentrate diet (feedlot ration) to lambs for six weeks with two weeks of adaptation [12] increased the oxidative stress in lambs as assessed by the blood biomarker, isoprostanes, as opposed to lambs fed a pasture diet (392 vs. 275 pg/mL for feedlot vs. pasture). The significant increase in blood isoprostanes concentration was highly and positively related to muscle LA (140 vs. 96 mg/kg muscle for feedlot vs. pasture, *p* < 0.001) and total n-6 (180 vs. 130 mg/kg muscle for feedlot vs. pasture) concentrations. This in turn was significantly and positively related to lipid oxidation (TBARS 5.4 vs. 3.5 for feedlot vs. pasture) in meat aged for 60 days at 2 °C and displayed for 72 h under simulated retail conditions [12].

Functional fatty acids such as omega-3 and omega-6 are stored in muscle cell membrane phospholipids that maintain membrane structure, fluidity and permeability [23]. Heat stress has been reported to activate muscle membrane phospholipases and phosphatidylinositol phosphate kinases within a short period of ambient temperature increase from 25–35 °C and above [24]. Our previous study revealed that significant changes in the physiological responses and dry matter intake occur when temperature exceeds 25 °C [25]. Elevated levels of thiobarbituric acid reactive substances (TBARS) such as malondialdehydes (MDA) were reported in poultry [26], buffalo [27] and dairy cattle [28], when animals were subjected to high environmental temperature. In the present study, the small increase in muscle omega-6 fatty acid (LA) with one week of exposure to HS did not affect lipid peroxidation of fresh and aged meat (stored for 42 days) displayed for 72 h as assessed by TBARS. However, we have noticed that, at a lower vitamin E concentration (2.95 mg/kg muscle) in muscle tissues from all treatments, lipid oxidation of fresh meat was not affected either at 1 h or 72 h (Day 2) display time. In the case of aged meat (stored for 42 days), the lipid oxidation was dramatically increased after 72 h of display time in lambs reared on CON and MOD diets, but the extent of lipid oxidation was significantly reduced in lambs fed the vitamin E diet at the SUP level.

Dietary supplementation of vitamin E to reduce lipid peroxidation and to enhance meat colour stability has been successfully used in the past [21,29]. It has been recently shown that vitamin E concentration has a predominant effect on lipid oxidation at levels above 2.95 mg/kg muscle irrespective of the level of PUFA or iron in the muscle [30]. However, it is important to note that vitamin E supplementation at levels of 250 IU/kg DM and 60 IU/kg DM is required to ensure enough vitamin E deposition in the muscle in lambs fed on concentrate based diets or green pasture based diets, respectively [31]. It is also known that to prevent lipid oxidation, a minimum of 1.9 mg/kg muscle of vitamin E is required. One week of heat stress or four weeks of vitamin E supplementation did not affect colour stability. However, there was a storage type effect over the 72 h display time, where the redness (a*-value > 14.3) or brownness formation (RF_630/580_ > 3.3) of fresh meat were within the accepted range for consumers [13]. With aged meat stored for 42 days and then displayed for 72 h duration, the redness and brownness formation ranges were below the acceptable range after 48 h of display. This implies that feeding systems using diets containing a high proportion of grains or in feedlots should be designed to maintain the vitamin E concentrations in muscle tissues above 3.0 mg/kg meat so that the deterioration of lipid and colour in meat stored for moderate and long term can be avoided. 

There are insufficient published data regarding the oxidative status of lambs finished during summer and influenced by heat stress compromising meat nutritional value and storage stability. This study provides some information that heat stress can alter the membrane lipid composition and that this may be pro-inflammatory, which in turn can lead to oxidative stress in animals. There is also evidence that oxidative stress induced by higher metabolic activities to support the growth rate and to defend against elevated environmental temperature can be reduced by higher dietary antioxidant feeding in finisher lambs. The major findings of this experiment were (1) antioxidant supplementation at the SUP level enhanced animal performance as observed by increased FLW, which in turn resulted in increased HCW compared with the MOD and CON groups; (2) short-term HS significantly increased muscle omega-6 fatty acid (linoleic acid) concentration compared with the TN group, believed to enhance pro-inflammatory actions through induced free radical formation and oxidative stress; (3) SUP treatment also increased the storage stability of meat as observed by delayed lipid oxidation in aged meat compared with other dietary treatments. Future studies are warranted to examine the impact of fluctuating daily night and day environmental temperatures on growth performance, carcass weight at slaughter and the nutritional quality of products post-farm from animals reared under extensive systems.

## 5. Conclusions

Results indicate that four weeks of antioxidant (vitamin E and Se) supplementation in the feedlot diet tended to increase muscle vitamin E concentration compared with a control feedlot diet. Antioxidant supplementation in the diet at the supranutritional level also increased final liveweight and hot carcass weight and reduced the lipid oxidation of aged meat when compared with lambs supplemented with the moderate or control antioxidant diets. Muscle linoleic and total n-6 fatty acid concentrations increased significantly following exposure to one week of heat stress, but this did not alter the muscle vitamin E concentration, lipid oxidation or retail colour stability of meat compared to the control group. Storage of meat for six weeks significantly reduced the lipid oxidative stability and colour stability of meat, below the threshold for consumer acceptance, which is believed to be because of insufficient muscle vitamin E concentration.

## Figures and Tables

**Table 1:** Effect of 4 weeks of dietary antioxidant (vitamin E and selenium) supplementation on carcass characteristics and muscle vitamin E concentration in finisher lambs exposed to one week of heat stress.

Items	Thermoneutral (TN)			Heat Stress (HS)			SED	*p*-Value		
	CON	MOD	SUP	CON	MOD	SUP	(D × HS)	Diet (D)	HS	D × HS
Initial liveweight (kg)	41.9	43.3	42.3	42.9	42.3	41.4	1.29	0.58	0.74	0.46
Final liveweight (kg)	44.2	44.1	46.3	44.6	43.5	47.3	1.77	0.05	0.77	0.83
Hot carcass weight (kg)	21.2	21.2	22.7	21.4	20.7	22.4	0.75	0.01	0.70	0.74
GR fat depth (mm)	9.67	7.91	9.75	9.92	8.00	10.20	0.98	0.01	0.63	0.96
Muscle 24 h pH	5.66	5.64	5.60	5.60	5.59	5.58	0.05	0.81	0.31	0.77
Muscle Vit. E (mg/kg)	1.75	1.92	1.96	1.72	2.13	2.07	0.25	0.15	0.50	0.79

Experimental diets: CON = control, MOD = moderate, SUP = supranutritional, SED = standard error of difference, GR = total tissue thickness over the 12th rib, 110 mm from the backbone, Vit E = vitamin E.

**Table 2:** Muscle (longissimus lumborum) fatty acid composition (mg/100 g muscle) of lambs finished on 4 weeks of experimental diets followed by one week of thermal treatment.

Items	Thermoneutral (TN)			Heat Stress (HS)			SED	*p*-Value		
	CON	MOD	SUP	CON	MOD	SUP	(D × HS)	Diet (D)	HS	D × HS
C10:0	4.8	4.8	4.7	5.5	5.5	4.3	0.82	0.43	0.44	0.59
C12:0	5.9	5.5	5.2	6.4	6.5	4.8	1.17	0.32	0.60	0.69
C14:0	97.9	96.1	91.4	116	108	86.8	17.0	0.31	0.38	0.61
C14:1	2.9	2.9	2.4	3.5	3.3	2.5	0.64	0.20	0.30	0.81
C15:0	10.6	10.9	10.9	13.4	12.3	10.8	1.74	0.65	0.19	0.50
C16:0	710	716	719	864	806	690	98.6	0.49	0.22	0.42
C16:1	44.6	45.8	41.3	54.1	48.6	41.1	6.74	0.22	0.31	0.58
C18:0	496	501	564	607	660	534	77.2	0.91	0.30	0.44
C18:1n-9*cis*	1204	1209	1233	1456	1345	1241	165	0.73	0.18	0.58
C18:2n-6 (LA)	87.3	88.6	81.1	97.1	89.2	89.8	4.95	0.17	0.03	0.37
C18:3n-3 (ALA)	32.8	33.8	33.1	35.6	36.0	34.4	4.21	0.93	0.38	0.97
C20:4n-6 (AA)	17.0	16.8	16.7	17.3	17.5	17.6	1.15	1.00	0.37	0.93
C20:5n-3 (EPA)	11.7	12.8	12.9	12.0	12.1	13.0	1.22	0.42	0.92	0.79
C22:5n-3 (DPA)	11.6	12.6	12.8	12.4	12.3	12.5	0.68	0.43	0.92	0.41
C22:6n-3 (DHA)	4.1	4.4	3.6	4.2	4.4	4.4	0.45	0.34	0.22	0.37
Total n-6	109	110	103	119	111	112	4.91	0.16	0.02	0.34
Total n-3	60.4	60.9	62.6	64.5	61.5	64.7	4.74	0.93	0.65	0.61
Ratio n-6:n-3	1.85	1.77	1.65	1.87	1.85	1.78	0.15	0.40	0.39	0.89
Total muscle fat	2790	2809	2884	3363	3115	2839	363	0.71	0.19	0.49

Experimental diets: CON = control, MOD = moderate, SUP = supranutritional.
